# Trade-Off Analysis of Classical Machine Learning and Deep Learning Models for Robust Brain Tumor Detection: Benchmark Study

**DOI:** 10.2196/76344

**Published:** 2025-09-15

**Authors:** Yuting Tian

**Affiliations:** 1 Thayer School of Engineering Dartmouth College Hanover, NH United States

**Keywords:** machine learning, deep learning, self-supervised learning, computer vision, brain tumor image

## Abstract

**Background:**

Medical image analysis plays a critical role in brain tumor detection, but training deep learning models often requires large, labeled datasets, which can be time-consuming and costly. This study explores a comparative analysis of machine learning and deep learning models for brain tumor classification, focusing on whether deep learning models are necessary for small medical datasets and whether self-supervised learning can reduce annotation costs.

**Objective:**

The primary goal is to evaluate trade-offs between traditional machine learning and deep learning, including self-supervised models under small medical image data. The secondary goal is to assess model robustness, transferability, and generalization through evaluation of unseen data within- and cross-domains.

**Methods:**

Four models were compared: (1) support vector machine (SVM) with histogram of oriented gradients (HOG) features, (2) a convolutional neural network based on ResNet18, (3) a transformer-based model using vision transformer (ViT-B/16), and (4) a self-supervised learning approach using Simple Contrastive Learning of Visual Representations (SimCLR). These models were selected to represent diverse paradigms. SVM+HOG represents traditional feature engineering with low computational cost, ResNet18 serves as a well-established convolutional neural network with strong baseline performance, ViT-B/16 leverages self-attention to capture long-range spatial features, and SimCLR enables learning from unlabeled data, potentially reducing annotation costs. The primary dataset consisted of 2870 brain magnetic resonance images across 4 classes: glioma, meningioma, pituitary, and nontumor. All models were trained under consistent settings, including data augmentation, early stopping, and 3 independent runs using the different random seeds to account for performance variability. Performance metrics included accuracy, precision, recall, *F*_1_-score, and convergence. To assess robustness and generalization capability, evaluation was performed on unseen test data from both the primary and cross datasets. No retraining or test augmentations were applied to the external data, thereby reflecting realistic deployment conditions. The models demonstrated consistently strong performance in both within-domain and cross-domain evaluations.

**Results:**

The results revealed distinct trade-offs; ResNet18 achieved the highest validation accuracy (mean 99.77%, SD 0.00%) and the lowest validation loss, along with a weighted test accuracy of 99% within-domain and 95% cross-domain. SimCLR reached a mean validation accuracy of 97.29% (SD 0.86%) and achieved up to 97% weighted test accuracy within-domain and 91% cross-domain, despite requiring 2-stage training phases involving contrastive pretraining followed by linear evaluation. ViT-B/16 reached a mean validation accuracy of 97.36% (SD 0.11%), with a weighted test accuracy of 98% within-domain and 93% cross-domain. SVM+HOG maintained a competitive validation accuracy of 96.51%, with 97% within-domain test accuracy, though its accuracy dropped to 80% cross-domain.

**Conclusions:**

The study reveals meaningful trade-offs between model complexity, annotation requirements, and deployment feasibility—critical factors for selecting models in real-world medical imaging applications.

## Introduction

Brain tumors, characterized by the abnormal growth of brain cells, pose significant health risks and can result in severe neurological dysfunction or death if not detected early [1,2]. Timely and accurate diagnosis is essential for effective treatment and improved patient outcomes [3]. Magnetic resonance imaging (MRI) is the widely used modality for capturing high-resolution brain images. However, the manual review of hundreds of MRI scans to identify tumors is time-consuming and error-prone, posing a considerable challenge for radiologists [3]. In this context, machine learning and deep learning approaches have demonstrated promising potential in improving diagnostic accuracy and efficiency [3].

Recent studies have increasingly focused on leveraging machine learning and deep learning for medical image classification tasks, including brain tumor detection [3-5]. However, selecting the appropriate type of model for small medical image datasets remains a challenge.

To address this challenge, we conducted a comparative analysis of 2 publicly and independent available MRI brain tumor datasets; our study included 1 classical machine learning model—support vector machine (SVM) with histogram of oriented gradients (HOG) features [6,7], and 3 deep learning models—convolutional neural network (CNN) based on ResNet18 [8], a vision transformer (ViT) based on ViT-B/16 [9], and a self-supervised learning (SSL) model based on Simple Contrastive Learning of Visual Representations (SimCLR) [10]. These models were selected to represent 4 distinct paradigms in medical image classification. SVM+HOG represents traditional feature engineering approaches with low computational requirements. ResNet18, a well-established CNN, is known for its strong baseline performance in small- to medium-sized datasets. ViT-B/16 introduces global attention mechanisms capable of capturing long-range dependencies. SimCLR enables representation learning from unlabeled data, offering potential benefits in annotation-scarce clinical settings. Each type of model offers unique advantages and limitations when applied to small medical image datasets [11], making them ideal for a trade-off comparative analysis.

To assess the applicability of trade-offs in small medical image datasets, five analytical perspectives were considered: (1) training behavior and convergence [12], (2) train and validation data performance [13], (3) generalization: within- and cross-domains [14], (4) visual interpretation via saliency maps [15], and (5) real-world cost, complexity, and deployment feasibility [11,16].

This comparative study aims to provide practical insights for both machine learning researchers and health care practitioners by identifying reliable, scalable, and computationally efficient artificial intelligence (AI) solutions for medical imaging applications.

## Methods

### Study Overview

This study investigates the trade-offs between traditional machine learning and deep learning approaches, including SSL, for brain tumor classification. The analysis was conducted using a primary medical imaging dataset [3,11]. To evaluate robustness and generalization capabilities, the models were assessed on unseen test data under both within-domain and cross-domain scenarios, simulating real-world deployment conditions.

### Dataset Description

The primary dataset used for training and validation is the Brain Tumor MRI Image Dataset (T1-weighted, 2D), an open-source resource hosted on Figshare [17]. It comprises a total of 2870 magnetic resonance images, categorized into 4 subsets: glioma (826 images), meningioma (822 images), pituitary (827 images), and no tumor (395 images).

To assess cross-domain generalization, we used a cross dataset of brain tumor magnetic resonance images (T1-weighted, 2D) compiled from multiple publicly available repositories, including Kaggle and Roboflow [18]. While the original dataset contains a mixture of computed tomography and MRI scans, only the MRI subset was retained for evaluation in this study. This cross dataset introduced natural domain shifts due to variations in image size, patient demographics, and file formats (eg, JPG and JPEG), thereby providing a realistic benchmark for evaluating generalization performance.

### Data Processing

The primary image dataset was partitioned into training, validation, and test sets. Within each partition, images were further organized into “tumor” and “no tumor” categories. Tumor (2475 images) was split into training, validation, and test datasets in a 70:15:15 ratio, while no tumor (395 images) was split using the same proportion.

The cross dataset was originally organized into “healthy” and “tumor” folders; these were relabeled as “no tumor” and “tumor,” respectively, to maintain consistency with the structure of the primary dataset. To prevent data leakage and ensure unbiased evaluation of generalization performance, the phash algorithm [19] was applied to compare each cross-dataset image with the training images; any visually identical or nearly identical images were removed from the cross dataset. After duplication, 3351 cross-dataset magnetic resonance images remained, including 2123 tumor and 1228 no tumor. All images were mapped to binary labels: 1 for “tumor” and 0 for “no tumor” cross dataset.

### Model Designing

#### Support Vector Machine + Histogram of Oriented Gradients

We applied an SVM classifier in combination with HOG feature extraction to classify brain tumor images. HOG was used to extract edge and shape information by analyzing the distribution of gradient orientations across localized regions of the image [6]. Because HOG effectively captures fine-grained structural patterns, it is particularly suitable for identifying tumor boundaries, which exhibit strong and localized gradient variations.

Following HOG-based feature extraction, the resulting feature vectors were fed into an SVM, a supervised learning algorithm widely recognized for its effectiveness in binary classification tasks [20]. In this study, we used a linear kernel since tumor and nontumor images exhibit distinct and approximately linearly separable gradient patterns in the HOG feature space [7].

#### Convolutional Neural Network (ResNet18)

A CNN based on ResNet18 was applied for brain tumor image classification. ResNet18 is a widely used deep learning architecture that integrates residual learning to mitigate the vanishing gradient problem [8]. It comprises 17 convolutional layers followed by a fully connected classification layer. Compared to traditional CNN models, the key advantage of ResNet18 lies in its use of 4 residual blocks, each consisting of 2 convolutional layers combined with batch normalization and rectified linear unit activation. Residual connections directly add the input of a block to its output, enabling the network to learn residual mappings and maintain a strong gradient flow during backpropagation [8,12] and mitigating the vanishing gradient issue. This feature makes ResNet18 particularly suitable for complex medical image tasks [21].

The residual block follows the logic: *y*=*F*(*x*)+*x*, where *F*(*x*) represents the convolutional transformation applied to the input *x*. The term *x* is passed directly to the next layer through a skip connection, allowing the network to learn residual mappings and maintain effective gradient flow.

To improve model generalization and mitigate overfitting, data augmentation techniques were applied during the training process [22]. These argumentations included random affine transformations with shear up to ±5 degrees, random scaling between 95% and 105%, and small random rotation up to ±3 degrees. In addition, 50% (n=866) of images were randomly flipped horizontally and 30% (n=520) vertically, and a Gaussian blur was applied using a kernel size of 3 with a sigma value randomly selected from the range 0.1 to 1.0. Furthermore, the images were resized on the shortest side to 224 pixels, followed by a center crop to ensure a consistent input size of 224×224. Finally, the images were converted to a tensor and normalized to pixel intensity values, using a mean of 0.5 (SD 0.5).

The validation data were preprocessed with minimal transformations, including resizing, center cropping, tensor conversion, and normalization with the same parameters. For fine-tuning, all pretrained layers except the last 3 residual blocks and the fully connected layer were frozen; these 4 layers were fine-tuned to adapt to the binary classification task. The fully connected layer was modified with randomly incorporating a 40% dropout rate, followed by a linear layer with 2 outputs corresponding to the tumor and no-tumor classes.

#### Vision Transformer (Vit-B/16)

ViT models are widely used in computer vision by leveraging self-attention mechanisms to capture long-range dependencies across an entire image [9]. Unlike CNNs, which learn hierarchical feature representations via local receptive fields, ViTs partition an image into nonoverlapping patches and process them as flattened tokens [9]. Those tokens are then passed through multiple transformer encoder layers, where multihead self-attention models both local and global relationships within the image.

In this study, we fine-tuned ViT-B/16 model by unfreezing the last 5 transformer encoder layers along with the classification head to better capture the complex long-range dependencies. ViT-B/16 comprises 12 self-attention heads with a hidden dimension of 768 [9]. Within each encoder layer, the feed-forward network includes 2 fully connected layers with Gaussian error linear unit activations, expanding the hidden dimension from 768 to 3072 before projecting it back to 768 [23].

#### Self-Supervised Learning (SimCLR)

SSL enables models to learn robust feature representations directly from unlabeled data. In this study, we applied SimCLR, which learns meaningful feature representations by maximizing agreement between different augmented views of the same image (positive pairs) while minimizing similarity between views of different images (negative pairs) through a contrastive loss in the latent space [10]. This approach enables the encoder to extract invariant and discriminative features, which are subsequently leveraged for downstream classification tasks [24].

During the pretrained phase, we applied various augmentations to generate 2 augmented views of the same image, denoted as *x_i_* and *x_j_*. These augmented images were then passed through a ResNet18-based encoder to extract feature representations. The embeddings were mapped into a lower-dimensional space using a projection head, which minimized the distance between the positive pairs, *x_i_* and *x_j_*, while maximizing the similarity from all other views using contrastive loss (normalized temperature-scaled cross entropy loss) [25].

Following pretraining, we performed a linear evaluation: the encoder was frozen, and a fully connected linear layer classifier was trained on top using supervised labels to evaluate the learned representations.

### Model Training

#### Support Vector Machine + Histogram of Oriented Gradients

The SVM+HOG model was trained and validated over 3 independent runs using different random seeds to ensure robustness. For each run, the training and validation datasets were first loaded and shuffled, and then, HOG was applied to each image after resizing to 128×128 pixels. The images were divided into small cells with 16×16 pixels, and a histogram of gradient orientations was computed per cell. Histograms were normalized across blocks with 2×2 cells to improve contrast robustness [6]. The resulting HOG feature vector was flattened into a 1D array with a length of 1764 features (*f*_1_, *f*_2_, ... *f*_1764_), representing the gradient information distribution captured from different parts of the image, preserving local shape information. No channel conversion was needed, as these tumor images were in grayscale format.

A support vector classifier from scikit-learn was used, with the default regularization parameters (*C*=1.0) and hinge loss [26]. Performance was evaluated on a validation dataset after each run. The training process was repeated across 3 independent runs, and accuracy was recorded for each run. The model with the highest validation accuracy across the 3 runs was selected and saved for testing.

#### Convolutional Neural Network (ResNet18)

The ResNet18 model was trained using Adam optimizer with a learning rate of 1×10^–4^ and a weight decay of 1×10^–5^; the classification task was optimized using a cross-entropy loss function, with labeling smoothing set to 0.05 [27,28]. To dynamically adjust the learning rate, a ReduceLROnPlateau scheduler was applied, which reduced the learning rate by a factor of 0.5 if the validation loss failed to improve over 3 consecutive epochs [29]. To prevent overfitting, early stopping was used: training terminated if validation loss failed to improve by at least 0.001 over 4 consecutive epochs. The model was trained for up to 35 epochs with a batch size of 32.

Model performance was monitored at each epoch, and the best-performing checkpoint based on the lowest validation loss from each run was saved. To ensure robustness and reproducibility, training was repeated across 3 independent runs with different random seeds. The results were reported as the mean and SD of accuracy and loss across the best-performance models from each run. After completing all 3 runs, the model with the best validation loss across runs was selected for evaluation on the unseen test data.

#### Vision Transformer (Vit-B/16)

For the ViT-B/16 model, we adopted the same data augmentation strategy used for ResNet18 to maintain experimental consistency. Due to ViT-B/16’s higher parameter count, the learning rate was increased to 3×10^–4^ to accelerate convergence [9]. The CosineAnnealing scheduler was used instead of ReduceLROnPlateau to achieve smoother decay, which is typically advantageous for transformer architectures [30]. The model was trained for up to 50 epochs, with early stopping patience set to 6 and batch size set to 32. Training was repeated 3 times with different seeds. The best checkpoint per run based on validation loss was saved, and the overall best-performing model was selected for testing.

#### Self-Supervised Learning (SimCLR)

SimCLR was pretrained to learn image representations using contrastive learning [10]. The ResNet18-based encoder produced a 512-dimensional embedding vector, which was then passed through a projection head consisting of 2 linear layers and rectified linear unit activation, reducing the vector to 128 dimensions. Pretraining used Adam optimizer, learning rate 1×10^–4^, weight decay 1×10^–5^, a batch size of 128, and up to 200 epochs. The Data Loader was configured with num_works equal to 4 to parallelize data loading and accelerated training. A ReduceLROnPlateau scheduler and early stopping were used [29]. After pretraining, the encoder was frozen, and a linear classifier was trained for 50 epochs with a batch size of 32. Training was repeated 3 times with different seeds, and the best checkpoint per run was saved. The overall best-performing model across runs was used for evaluation on the unseen test dataset.

### Model Evaluation

To ensure a fair and reproducible comparison across all models, we evaluated their performance from 5 perspectives: training behavior and convergence, training and validation data performance, generalization: within- and cross-domains, visual interpretation via saliency maps, and real-world cost, complexity, and deployment feasibility.

For training behavior and convergence, we monitored epoch-wise training and validation accuracy and loss. Because early stopping caused different runs to terminate at varying epochs, shorter training runs were padded to match the maximum number of epochs while excluding padded values from the statistics. This approach enabled a consistent assessment of convergence stability across models.

For training and validation data performance, we compared training and validation accuracy, training and validation loss, and training time across models on the primary dataset. Accuracy and loss were computed on both the training and validation sets to assess potential overfitting and robustness. Training time was also recorded to evaluate computational efficiency. Furthermore, classification performance on the validation data was assessed using precision, recall, *F*_1_-score, and support for each class (tumor and nontumor), along with macro and weighted averages and overall accuracy.

For generalization within- and cross-domains, we assessed classification performance and confusion matrices on the unseen test datasets from both the primary and cross-domains. To better simulate real-world conditions, no test-time augmentations or artificial noise were applied during evaluation. To ensure reproducibility of the test-time results, all random seeds were fixed to 42 at the start of each model evaluation. Each model was evaluated using the best-performing weights obtained during training.

For visual interpretation via saliency maps, we examined model interpretability and generalization by visualizing the regions of the input images that most influenced classification decision. Saliency maps were generated for the same tumor and nontumor images from the primary dataset across all 4 models to ensure consistency in comparison. Similarly, 1 tumor and nontumor image from the cross dataset were used to examine how each model attended to relevant features across models.

Finally, for real-world cost, complexity, and deployment feasibility, we assessed each model’s computational resource requirements, training and inference efficiency, and practicality for integration into real-world clinical workflows.

### Ethical Considerations

This study did not involve human or animal participants. Institutional review board approval, informed consent, data confidentiality, and participant compensation were not applicable.

## Results

### Training Behavior and Convergence

**Figure 1:**
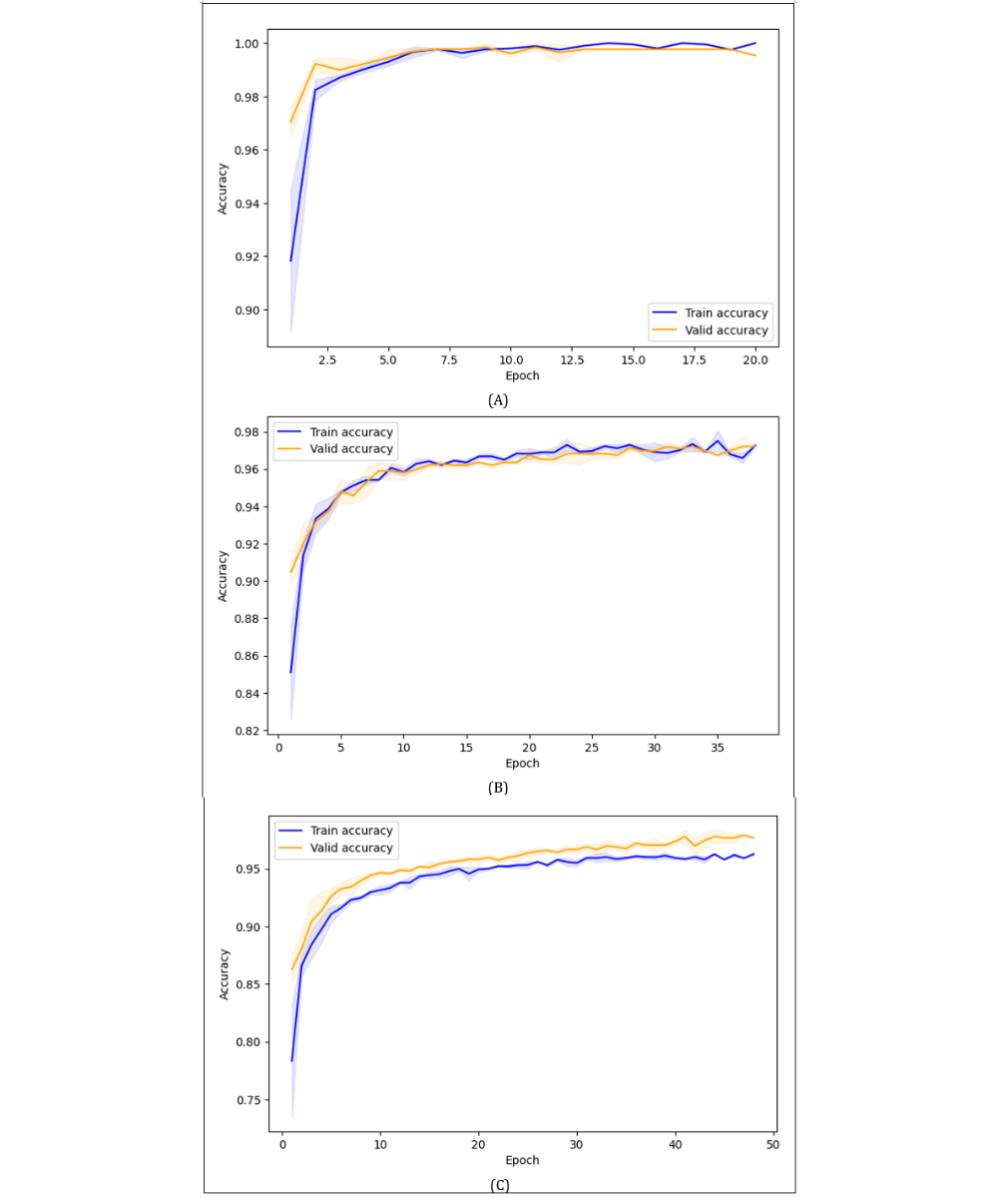
Training and validation accuracy curves for (A) ResNet18, (B) ViT-B/16, and (C) SimCLR. SimCLR: Simple Contrastive Learning of Visual Representations; ViT: vision transformer.

**Figure 2:**
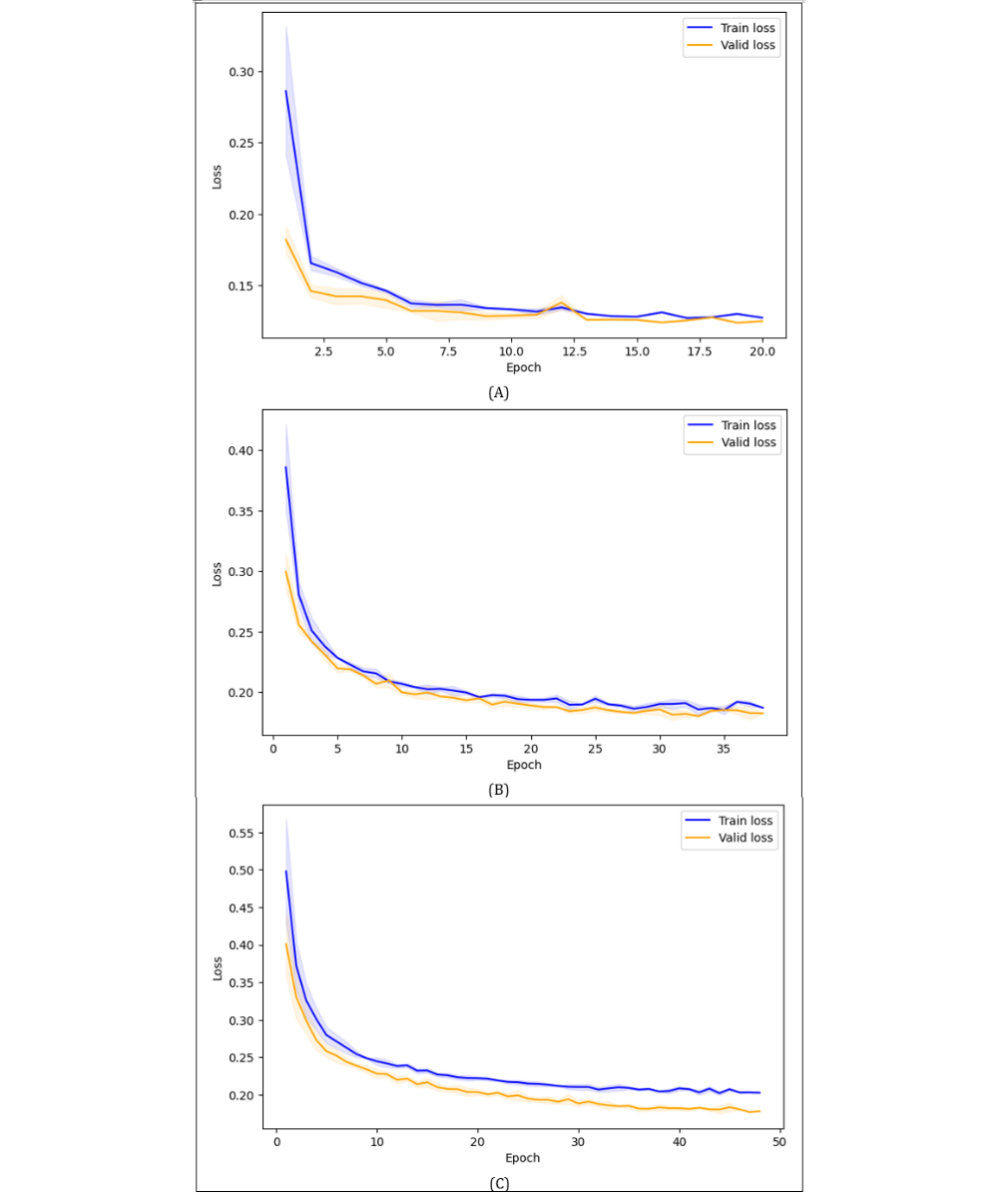
Training and validation loss curves for (A) ResNet18, (B) ViT-B/16, and (C) SimCLR. SimCLR: Simple Contrastive Learning of Visual Representations; ViT: vision transformer.

Figure 1 shows the training and validation accuracy across epochs for (A) ResNet18, (B) ViT-B/16, and (C) SimCLR, while Figure 2 presents the corresponding training and validation loss curves. Across the deep learning models, training and validation accuracy increased steadily during early epochs and gradually plateaued toward the later stages of training. Additionally, the training and validation curves remained closely aligned, with only small fluctuations, indicating stable learning progress. Similarly, training and validation loss decreased consistently over time, with validation loss typically remaining slightly lower or close to training loss. These trends demonstrated that ResNet18, ViT-B/16, and SimCLR achieved stable convergence without significantly overfitting or underfitting. The SVM+HOG does not have an epoch-based training process; hence, no convergence curve was shown.

Additionally, due to early stopping, different runs of the same deep learning models terminated at varying epochs, and the maximum number of training epochs observed across the 3 runs was 20 for ResNet18, 38 for ViT-B/16, and 48 for SimCLR.

Notably, across all deep learning models, training accuracy was slightly lower than validation accuracy, and training loss was slightly higher or close to the validation loss. This was due to the use of data augmentation during training, which introduced random transformations such as cropping, flipping, and Gaussian blur to improve generalization. These argumentations made the training more challenging, whereas validation data remained nonaugmented, leading to relatively easier and more consistent predictions.

### Training and Validation Data Performance

Table 1 summarizes the training and validation performance across 4 models. Among deep learning models, ResNet18 achieved the highest validation accuracy (mean 99.77%, SD 0.00%) with the lowest validation loss (mean 12.58%, SD 0.21%), demonstrated strong convergence and stability. ViT-B/16 and SimCLR also performed well, achieving mean validation accuracies of 97.36% (SD 11%) and 97.29% (SD 86%), respectively, though their slightly higher training and validation losses indicated slower convergence compared to ResNet18. For the classical model SVM+HOG, mean validation accuracy reached 96.51% (SD 0.00), indicating no overfitting or underfitting.

**Table 1:** Training and validation performance comparison across 4 models.

Model	Training time (seconds)	Training accuracy (%), mean (SD)	Validation accuracy (%), mean (SD)	Training loss (%), mean (SD)	Validation loss (%), mean (SD)
SVM^a^+HOG^b^	78	—^c^	96.51 (0.00)	—	—
ResNet18	529	99.80 (0.08)	99.77 (0.00)	13.25 (0.19)	12.58 (0.21)
ViT-B/16^d^	2014	97.05 (0.46)	97.36 (0.11)	19.12 (0.43)	17.97 (0.21)
SimCLR^e^	2923	96.02 (0.22)	97.29 (0.86)	20.49 (0.38)	17.84 (0.28)

^a^SVM: support vector machine.

^b^HOG: histogram of oriented gradients.

^c^Not available.

^d^ViT: vision transformer.

^e^SimCLR: Simple Contrastive Learning of Visual Representations.

Regarding computational efficiency, SimCLR required the longest training time (2817 seconds), due to its self-supervised pretraining and linear evaluation phases, followed by ViT-B/16 (912 seconds) and ResNet18 (474 seconds), while SVM+HOG trained extremely fast (75 seconds) but lacked fine-tuning capabilities. We used an NVIDIA RTX A6000 graphics processing unit (GPU) during training, so the runtime may vary depending on the reviewer’s GPU or central processing unit setup and use, despite consistent code and hyperparameters.

Detailed visual comparisons of training and validation accuracy, loss, and training time charts are provided in Multimedia Appendix 1. In the figure, the top bar chart shows training and validation accuracy across models, the middle bar chart shows the training and validation loss across models, and the bottom bar chart shows training time comparison across models.

Table 2 summarizes the validation classification table, showing strong and consistent performance across all 4 models. Because the primary brain tumor dataset was imbalanced, with 59 no tumor samples and 371 tumor samples, we focused on weighted average metrics, including precision, recall, and *F*_1_-score, which accounted for class imbalance by weighting results based on class size.

**Table 2:** Comparison of classification performance on the validation data.

Model	Precision (weighted average)	Recall (weighted average)	*F*_1_-score (weighted average)	Overall accuracy
SVM^a^+HOG^b^	0.96	0.97	0.96	0.97
ResNet18	1.00	1.00	1.00	1.00
ViT-B/16^c^	0.97	0.97	0.97	0.97
SimCLR^d^	0.98	0.98	0.98	0.98

^a^SVM: support vector machine.

^b^HOG: histogram of oriented gradients.

^c^ViT: vision transformer.

^d^SimCLR: Simple Contrastive Learning of Visual Representations.

ResNet18 achieved the highest overall performance, with 100% accuracy, 100% weighted precision, 100% weighted recall, and 100% weighted *F*_1_-score. SimCLR, ViT-B/16, and SVM+HOG also demonstrated strong performance, with weighted *F*_1_-scores ranging from 96% to 98%, precision between 96% and 98%, and recall between 97% and 98%. These results demonstrated strong discriminative ability across models on the validation data and established a solid baseline for subsequent evaluations on unseen test data within- and cross-domains.

For detailed classification table reports for all models on the primary domain validation sets, please refer to Multimedia Appendix 2. The figure presents classification metrics including precision, recall, *F*_1_-score, and support for each class (tumor and nontumor) across 4 models, along with macro and weighted averages and overall accuracy. ResNet18 achieved perfect classification performance, while ViT-B/16 and SimCLR showed similarly high results. SVM+HOG showed slightly lower performance, especially in recall for the nontumor class.

### Generalization: Within- and Cross-Domains

Table 3 summarizes the comparison of classification performance on the unseen test data within- and cross-domains, demonstrating the strong and consistent performance across all models for both primary and cross datasets.

**Table 3:** Comparison of classification performance on the test data within- and cross-domains.

Model and dataset			Precision (weighted average)		Recall (weighted average)		*F*_1_-score (weighted average)		Overall accuracy
**SVM^a^+HOG^b^**									
	Primary	0.97		0.97		0.97		0.97	
	Cross dataset	0.80		0.80		0.80		0.80	
**ResNet18**									
	Primary	0.99		0.99		0.99		0.99	
	Cross dataset	0.96		0.95		0.95		0.95	
**ViT-B/16^c^**									
	Primary	0.98		0.98		0.98		0.98	
	Cross dataset	0.93		0.93		0.93		0.93	
**SimCLR^d^**									
	Primary	0.97		0.97		0.97		0.97	
	Cross dataset	0.92		0.91		0.91		0.91	

^a^SVM: support vector machine.

^b^HOG: histogram of oriented gradients.

^c^ViT: vision transformer.

^d^SimCLR: Simple Contrastive Learning of Visual Representations.

Both test data have imbalanced class distributions, including 60 nontumor and 372 tumor cases in the primary dataset and 1228 nontumor and 2123 tumor cases in the cross dataset. We applied weighted average metrics to evaluate the performance of the classification results. Across both datasets, ResNet18 consistently outperformed the other models, achieving the highest overall accuracy of 99%, along with the best weighted precision, recall, and *F*_1_-score on the primary dataset. On the cross dataset, these metrics slightly dropped to 95%. ViT-B/16 dropped from 98% within-domain to 93% cross-domain, and SimCLR dropped from 97% to 91%.

In contrast, SVM+HOG showed a sharp performance drop, with overall accuracy, precision, recall, and *F*_1_-score decreasing from 97% on the primary dataset to 80% on the cross dataset. This decline indicated SVM+HOG’s limited ability to generalize to new domains, likely due to its reliance on hand-crafted features rather than deep learning representations.

Notably, the models’ training behavior showed no signs of overfitting or underfitting as observed in Figures 1 and 2, indicating that the performance dropped not due to poor generalization during the training process but rather due to domain shift, including differences in patient demographics, imaging conditions, and equipment between the primary and cross datasets. Overall, ResNet18, ViT-B/16, and SimCLR maintained strong performance, demonstrating robust generalization within- and cross-domains, whereas SVM+HOG showed limited generalization under domain shift.

The detailed classification table reports for all models on the primary domain test sets and the cross-domain test sets are present in Multimedia Appendices 3 and 4, respectively.

Multimedia Appendix 3 represents classification metrics including precision, recall, *F*_1_-score, and support for each class (tumor and nontumor) on the test set across 4 models, along with macro and weighted averages and overall accuracy on the primary domain. ResNet18 also achieved the highest metrics overall, with 99% overall accuracy. ViT-B/16 followed closely, with a weighted average of 98% across precision, recall, and *F*_1_-score. SVM+HOG also performed well in the test data, with an overall accuracy of 97%, but slightly dropped on precision and *F*_1_-score of the nontumor class. SimCLR achieved a weighted average of 97% across metrics, still indicating strong performance on the primary dataset.

Multimedia Appendix 4 represents classification metrics including precision, recall, *F*_1_-score, and support for each class (tumor and nontumor) across 4 models, along with macro and weighted averages and overall accuracy on the cross-domain. ResNet18 remained the best performer, achieving a weighted precision, recall, and *F*_1_-score of 96% and an overall accuracy of 95%. ViT-B/16 followed with a weighted average of 93% for all metrics. SimCLR showed a moderate drop. SVM+HOG, with all weighted metrics, dropped to 80%, especially the lower recall on the nontumor class with 64%. These results indicated the strong robustness of deep learning models.

**Figure 3:**
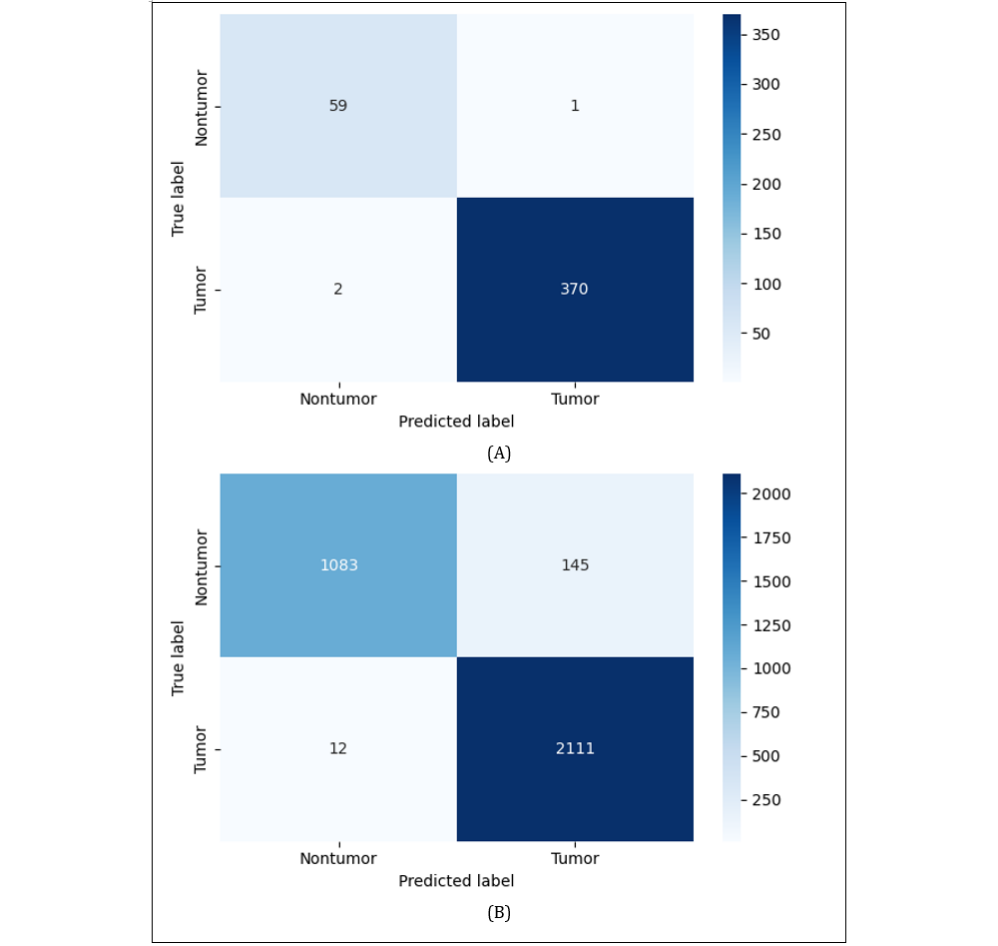
The confusion matrices of the ResNet18 evaluation on the unseen test set from the (A) primary domain and (B) cross-domain.

Figure 3 shows the confusion matrices of the ResNet18 evaluation on test sets from both the (A) primary domain and (B) cross-domain. On the primary domain, ResNet18 achieved highly reliable predictions, correctly identifying 370 tumors and 59 nontumors, with only 3 misclassified samples. However, on the cross dataset, the number of false positive cases increased sharply to 145 nontumor images that were incorrectly predicted as tumor images, while false negative cases remained low, with 12 tumor images being mis-predicted as nontumor images. This discrepancy may reflect shifts in data distribution that the cross dataset was not exposed during the training and validation process, and the model was trained and validated only on the primary dataset. Therefore, the cross dataset may contain different imaging conditions, such as brightness, noise level, and patient demographics, which can affect the appearance of nontumor brain structures and could cause nontumor images to be overdiagnosed as tumor images, even though the model’s training behavior showed no signs of overfitting or underfitting.

To conserve space and avoid redundancy, we presented only the confusion matrices on unseen test data of the best performing on ResNet18 across both the primary and cross datasets in the main text, while other confusion matrices for other models are presented in Multimedia Appendix 5 (SVM+HOG), Multimedia Appendix 6 (ViT-B/16), and Multimedia Appendix 7 (SimCLR).

Multimedia Appendix 5 presents the confusion matrices of the SVM+HOG model evaluated on unseen test data from the (A) primary and (B) cross datasets. On the primary dataset, the model correctly classified 54 nontumors and 365 tumor images, achieving good performance. However, its generalization cross-domains was limited, and on the cross dataset, performance dropped to 80% overall accuracy, and the number of false positives increased to 436 and false negatives increased to 226. This indicated that SVM+HOG performed well within the primary domain but more sensitive to domain shift compared to deep learning models, likely due to its reliance on hand-crafted features.

Multimedia Appendix 6 presents the confusion matrices of the ViT-B/16 model evaluated on unseen test data from the (A) primary and (B) cross datasets. ViT-B/16 achieved strong classification performance on both the primary and cross datasets, though some domain shift was observed. The primary dataset performed well with minimal false counts, and on the cross dataset, the performance slightly dropped, with an overall high accuracy of 93%, while the output retained high sensitivity tumor detection. The increase in false positives indicated some sensitivity to domain shift. Generally, ViT-B/16 generalized reasonably well cross-domain.

Multimedia Appendix 7 presents the confusion matrices of the SimCLR model evaluated on unseen test data from the (A) primary and (B) cross datasets. SimCLR showed solid classification performance on the primary and cross datasets. However, on the cross dataset, the number of false positives increased and indicated that SimCLR’s generalization ability is more sensitive to domain shifts compared to other deep learning models.

### Visual Interpretation via Saliency Maps

To better understand how each model identifies tumor-related feature, we applied visual interpretation techniques on selected images from both the primary and cross datasets. Saliency maps were generated to highlight regions that are most influential to model predictions. As SVM+HOG is a nondifferentiable model, we did not apply gradient-based saliency map [15], as we used for ResNet18 and SimCLR models, or attention rollout [31], as we used for ViT-B/16 model. Instead, we applied occlusion sensitivity [14] analysis for generating saliency maps on both tumor and nontumor images.

On the primary dataset, we selected 1 tumor image “m1 (160).jpg” and 1 nontumor image “image (28).jpg.” On the cross dataset, we chose 1 tumor image “tumor (49).jpg” and 1 nontumor image “mri_healthy (1853).jpg.” These 4 images were unseen test images, and their predicted labels match the true labels.

**Figure 4:**
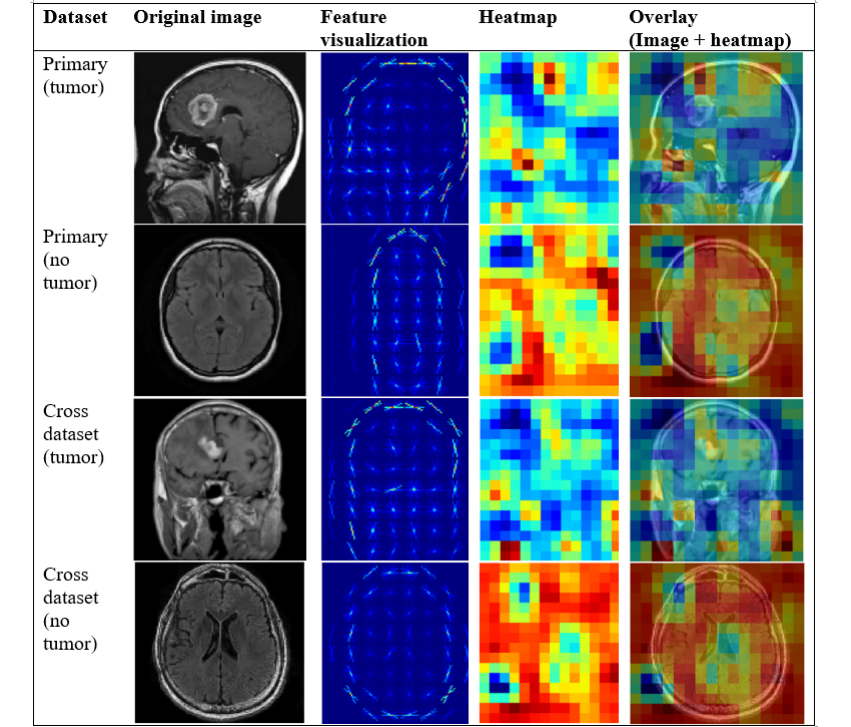
Visualization of SVM+HOG model features and occlusion sensitivity maps. Each row showed an example from the primary or cross dataset, categorized by tumor or nontumor class. The four columns displayed (1) the original magnetic resonance image, (2) the featural visualization, (3) the computed heatmap, and (4) the overlay of the heatmap on the original image. HOG: histogram of oriented gradients; SVM: support vector machine.

Figure 4 shows the visualization of SVM+HOG model features and occlusion sensitivity maps. We applied occlusion sensitivity [14] analysis to occlude patches of the input image to evaluate the drop in the SVM’s prediction probability, indicating the importance of each region for the classification prediction. In the overlay image, localized red or yellow regions indicated high sensitivity, which means occluding these regions significantly reduced the predicted probability of the tumor class. Blue and green regions indicated low sensitivity, which means occluding these regions had little or no impact on the model’s prediction.

For the tumor in the overlay image, on the primary dataset, we observed bright activations inside the brain area on the top left range, which partially coincided with visible anomalies in the original image. However, we also observed activations along the skull and outside of the brain area, which are unlikely to have clinical meaning and may reflect spurious features learned from the noise. On the cross dataset, we observed that there were localized bright spots in the top middle region inside of brain, indicating that the model may have partially identified a relevant region; however, there was still considerable noise presented along the skull and outside the brain part. Given the simplicity and limits of SVM+HOG features, such alignments may not be accurate and reliable.

For the nontumor images, both on the primary and cross datasets, the red or yellow regions were dispersed across the images, indicating that the model is less confident and possibly focusing on nondiagnostic features.

**Figure 5:**
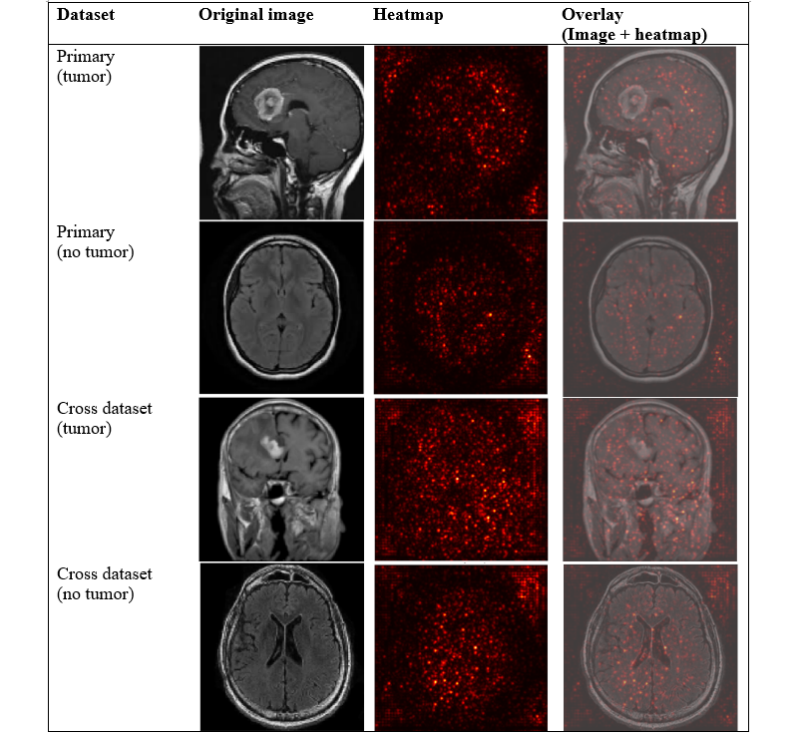
Gradient-based saliency maps for ResNet18 model. Each row showed an example from the primary or cross dataset, categorized by tumor or nontumor class. The three columns displayed (1) the original magnetic resonance image, (2) the computed heatmap, and (3) the overlay of the heatmap on the original image.

Figure 5 shows the gradient-based saliency maps for ResNet18 [15]. This method calculated the gradient of the model’s output score with respect to each input pixel, allowing us to identify regions that most influence the prediction. This approach aligns with the underlying mechanism of CNNs, which learns hierarchical feature representations through differential layers. In the saliency map, bright red areas indicate regions where small pixel changes impact the model’s prediction.

Notably, the gradient-based saliency maps do not directly identify tumor locations but instead highlight regions that are influential to the prediction. In the primary tumor image, we observed localized red regions on the back side of the brain; however, the anomalous region was more likely located at the front side of the brain based on the original image. Additionally, in the external brain tumor, the red regions were less localized and less prominent. For both nontumor images, the red activations were sparse and not strongly localized.

**Figure 6:**
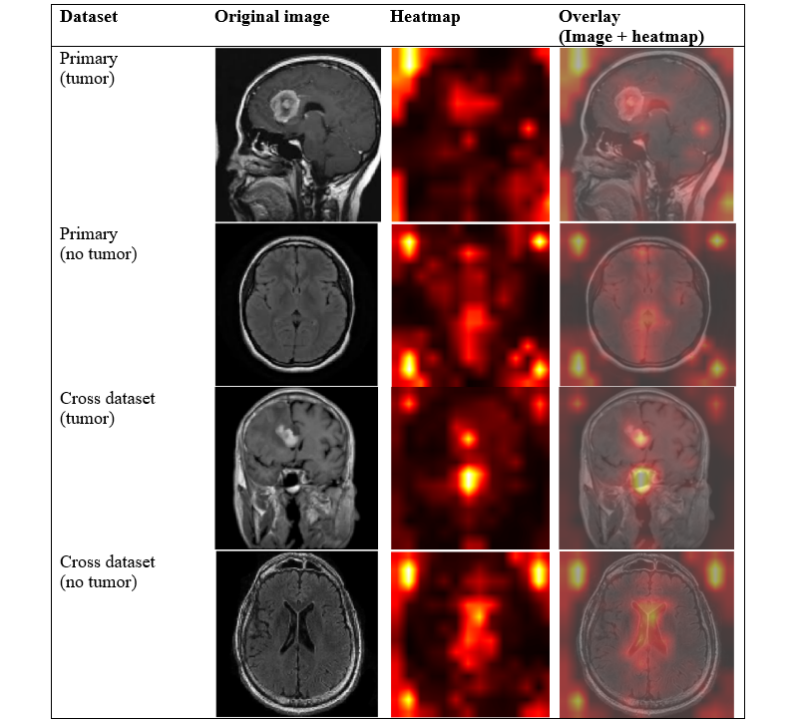
Attention-rollout saliency maps for ViT-B/16 model. Each row showed an example from the primary or cross dataset, categorized by tumor or nontumor class. The three columns displayed (1) the original magnetic resonance image, (2) the computed heatmap, and (3) the overlay of the heatmap on the original image. ViT: vision transformer.

Figure 6 shows attention-rollout [31] saliency maps to the ViT-B/16 model. This method aggregates attention weights across all transformer layers, representing how information flows through the network by propagating attention scores recursively. Specifically, it multiplies the attention matrices from different layers, effectively rolling out the attention signal to show which patches most influence the model classification prediction.

In the primary tumor image, red patches were concentrated in the front region of the brain, which well aligned with the visible anomaly in the original image, indicating that the model accurately identified the tumor location. In the external tumor image, the model also highlighted the central bright patches inside the brain, overlapping with the visible tumor, indicating a reasonable prediction. For both nontumor images, the patches were more diffusely spread, with no dominant hotspots; however, in the external nontumor image, slight attention near the ventricle area may reflect mild overattention to anatomical structure.

Compared to Figure 5 (ResNet18), the heatmaps from Figure 6 (ViT-B/16) appeared smoother and more globally distributed, reflecting the global attention mechanism to transformer models, in contrast to the more localized sensitivity observed in CNN-based saliency maps.

**Figure 7:**
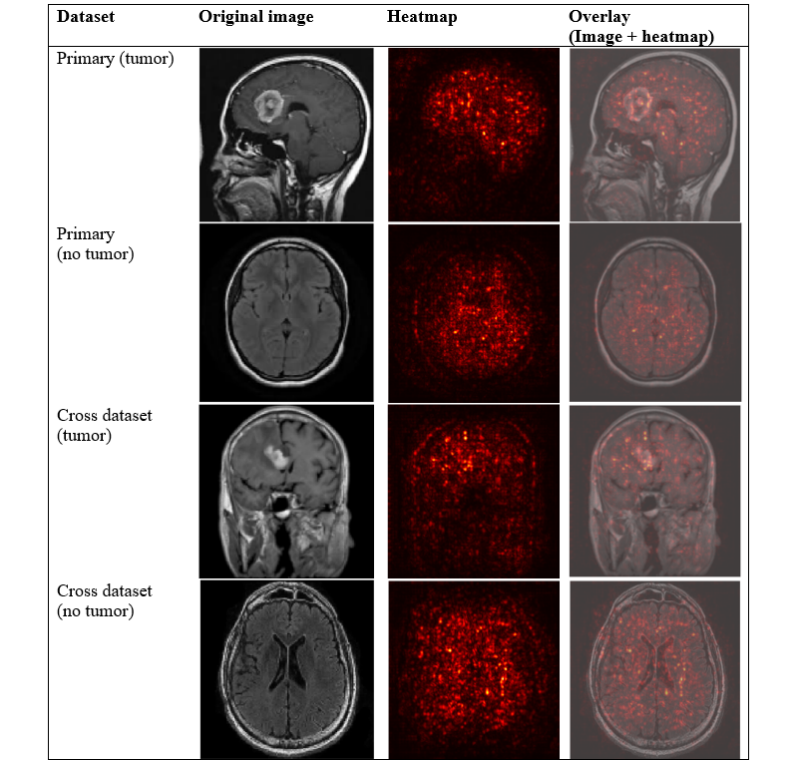
Gradient-based saliency maps for SimCLR model. Each row showed an example from the primary or cross dataset, categorized by tumor or nontumor class. The three columns displayed (1) the original magnetic resonance image, (2) the computed heatmap, and (3) the overlay of the heatmap on the original image. SimCLR: Simple Contrastive Learning of Visual Representations.

Figure 7 shows the gradient-based saliency maps [15], which visualize the regions that are most influential in SimCLR’s downstream classification. In the heatmaps, brighter red areas indicate pixels where small changes significantly influence the model’s prediction.

In both tumor and nontumor images, the saliency patterns were generally similar to those we observed in Figure 5 (ResNet18), though SimCLR’s saliency patterns appeared sharp and more diffuse. This is likely because SimCLR pretrained encoder learns from many augmented views without label annotation, which encourages the model to capture more global structure consistency rather than relying solely on highly localized features.

In the primary tumor image, we observed that there were bright red spots on the frontal region and the lower back region of the brain, partially aligning with the visible anomaly in the original image. In the external image, red spots were concentrated around the middle-left brain region, overlapping with the tumor area.

In both nontumor images, the saliency maps appeared as uniformly distributed, with no dominant regions. Notably, in both primary and cross-domain tumor images, SimCLR’s saliency maps displayed brighter and smoother red regions, with greater focus on the anomalous areas compared to those in Figure 5 (ResNet18), potentially making tumor regions more distinguishable in the saliency visualization.

Among the 4 saliency methods, attention rollout [31] from the ViT-B/16 model provided the most biologically meaningful explanations, with smoother and more globally coherent maps that aligned well with tumor regions in both the primary and cross datasets. SimCLR’s gradient-based [15] saliency showed better results, with clearer and more generalizable tumor region activations than ResNet18, likely due to its global feature learning through self-supervised contrastive pretraining. In contrast, SVM+HOG’s occlusion maps [14] were limited, interpretable, and often included spurious activations.

The interpretability of saliency maps does not always correlate with model performance. Gradient-based saliency maps from ResNet18 were less reliable and may not reflect biological meaningful reasoning, aligning with recent findings that question their trustworthiness in medical imaging [32]. Future work could explore more robust interpretability techniques to better uncover model behavior and support clinical decision-making.

### Real-World Cost, Complexity, and Deployment Feasibility

The SVM+HOG model is lightweight among these 4 models. It requires no GPU and has minimal memory use. It also requires grayscale images and simple feature extraction (HOG), making it ideal for low-resource settings. For instance, SVM+HOG could be deployed in community clinics or rural hospitals where computational infrastructure is limited, enabling rapid preliminary screening without the need for GPU resources.

The ResNet18 model balances moderate performance with reasonable efficiency, and it requires a GPU for fast training. It benefits from transfer learning by fine-tuning only the last few layers, making it more adaptable to small datasets. It is relatively compact in size and easily deployable on standard medical AI pipelines.

ViT-B/16, while achieving competitive accuracy, is the most computationally expensive, and it requires more memory, longer convergence, and benefits from larger datasets to reach optimal performance. These requirements may limit their feasibility in real time or low-resource clinical deployments.

SimCLR, with its 2-phase training, including pretraining and linear evaluation, is the most resource-intensive. Its deployment may be more complex due to the need for pretrained encoders and additional infrastructure for self-supervised pretraining. Despite the high initial cost, it shows strong generalization and can be highly effective when unlabeled data are abundant, but annotation resources are limited. For instance, SimCLR may be especially suitable for large medical centers with access to vast unlabeled image repositories but limited clinician time for annotation, allowing for scalable representation learning before fine-tuning.

While models like ViT-B/16 and SimCLR are more computationally demanding, their saliency visualizations provided more biologically meaningful explanations, which may support clinical trust and decision-making. In contrast, the simpler SVM+HOG model, although lightweight and suitable for low-resource settings, occasionally relied on spurious features, as reflected in its occlusion maps. The gradient-based saliency map on ResNet18 showed less reliability on medical imaging data.

## Discussion

### Principal Findings

The study presented a comparative trade-off analysis of 4 models, including SVM+HOG, ResNet18, ViT-B/16, and SimCLR, on 2 small-scale brain tumor MRI datasets. The evaluation spans multiple dimensions, including classification accuracy, robustness on the distribution shifts, generalization within- and cross-domains, training stability, and real-world deployment feasibility. Notably, the models demonstrated strong transferability and true generalization when tested on unseen data from an external independent dataset.

For a machine learning research perspective, model performance is typically prioritized. The results showed that ResNet18 delivered a strong balance between performance and training efficiency, making it suitable for settings where moderate resources are available. SimCLR offers compelling generalization with the advantage of requiring no labeled data during pretraining, making it well-suited for real-world conditions with annotation limitations [11]. ViT-B/16 shows potential, particularly due to its global attention mechanism, but may be more sensitive to overfitting under a small number of medical image datasets. SVM+HOG, although simpler, performs reliably with low computational cost and without relying on data augmentation [7].

For a clinical or radiologist perspective, model interpretability plays a critical role. In this regard, ViT-B/16 demonstrates the most coherent and biologically meaningful saliency maps through its attention-rollout mechanism, highlighting abnormal regions with greater reliability. SimCLR, despite its 2-stage training phases, produced saliency maps; these were smoother and more focused than those from ResNet18, likely due to its global feature learning through self-supervised contrastive training. Moreover, SimCLR requires no labeled data during pretraining, significantly reducing annotation effort for clinicians [24]. SVM+HOG, while occasionally producing spurious saliency activations under noise conditions, is an extremely lightweight and easy to deploy model. It does not require GPUs or deep learning architectures and can serve as a practical tool in resource-limited clinical environments or as a fast, interpretable baseline [33]. ResNet18 saliency map, relying on gradient-based method only, showing less reliable interpretability compared to ViT-B/16 and SimCLR, aligning with prior literature questioning the trustworthiness of gradient-based explanations in medical imaging [32].

### Hyperparameter Selection and System Setup

To ensure reproducibility and model optimization, we systematically tuned key hyperparameters across all models. The details tested ranges and final selected hyperparameters are provided in Multimedia Appendix 8, while a summary of the overall system configurations across the 4 models is provided in Multimedia Appendix 9.

Multimedia Appendix 8 summarizes the model optimization strategies. For ResNet18 and ViT-B/16, lower learning rates combined with weight decay were selected to stabilize training and prevent overfitting. CosineAnnealing LR was adopted for ViT-B/16 to handle its slower convergence, which ReduceLROnPlateau performed better for ResNet18. Data augmentation strategies, such as random rotations and affine transformations, improved robustness to geometric variations. For SimCLR, we optimized the projection head dimensions and augmentations to enhance representation learning. For SVM+HOG, a linear kernel and default regularization parameter provided the best trade-off between simplicity and performance.

Multimedia Appendix 9 summarizes the complete experiment environmental settings to ensure reproducibility. It detailed the hardware (NVIDIA RTX A6000 GPU, Intel Core i7 central processing unit, 251 GB RAM), operating system (Windows 11), and software frameworks (PyTorch 2.6.0+cu118 and torchvision 0.21.0+cu118). We also listed the Python (version 3.9.21; Python Software Foundation) and the key Python packages with their current versions, such as scikit-learn 1.6.1 and numpy 2.0.2. Finally, we set the random seed configuration used in the experiments; variable seeds were applied during training (42+run) to enhance model generalizability, while a fixed seed (42) was used during test runs to ensure deterministic evaluation results.

### Comparison With Previous Studies

Previous studies have explored various strategies for brain tumor classification, ranging from traditional machine learning models to deep learning approaches. One study [34] demonstrates that an SVM model alone as the baseline to attain the accuracy of 86.57% on unseen brain tumor data, incorporating principal component analysis improved the accuracy to 94.20%. While the combination of SVM with HOG and local binary pattern achieved a higher accuracy of 96.03%. Another study [35] applied ResNet18 for brain tumor detection and reported superior performance over models such as GoogLeNet and CapsNet, with their ResNet18 achieving an accuracy of 88.33%.

While previous studies primarily focused on classification accuracy, our research explored 4 distinct model mechanisms and interpreted how each identified image classification predictions based on learned features and internal decision processes. Our models demonstrated strong and consistent performance across multiple evaluation criteria.

Specifically, for the SVM+HOG model, the HOG method is used to extract edge and shape features from image data, which are then fed into an SVM for binary classification. ResNet18, a CNN model, predicts by attending to localized pixel patterns and patch movements. ViT-B/16, a transformer-based model, aggregates attention weights across all image patches and their spatial relationship, capturing global context for classification. SimCLR uses a pretrained ResNet18 as a backbone to learn localized feature representations through self-supervised contrastive learning, followed by a linear classifier for downstream prediction.

Additionally, we extended our evaluation to assess the robustness and generalization both within-domain and cross-domain. We analyzed training convergence patterns and included the practical feasibility metrics such as training time, annotation cost, and interpretability via saliency map. This comparative trade-off analysis is for offering a broader and practical evaluation framework for deploying AI in real-world medical imaging tasks.

### Limitations

This study has several limitations. First, the relatively small training images in the primary dataset contained 2008 images, and the cross-domain test data contained 3351 images. These dataset sizes were relatively small, particularly for deep learning models, which typically required large amounts of data to generalize better [3]. Second, we selected ResNet18, ViT-B/16, and SimCLR to enable fair baseline comparisons, but larger models such as ResNet50 or ViT-L may yield better performance. Third, we focused exclusively on image-level classification rather than voxel-level tumor localization. Finally, the interpretability of saliency maps does not always correlate with model performance and may potentially highlight spurious features.

### Future Work

For future work, larger and more diverse datasets are needed to validate model generalization and enhance clinical relevance. Extending this work to segmentation and 3D modeling could offer more precise tumor localization and enhance voxel-level clinical interpretation [36]. Moreover, exploring more robust interpretability techniques such as concept-based methods or counterfactual explanations may strengthen clinician trust in model decisions. Finally, close collaboration between machine learning researchers and clinicians will be critical to translating models into trustworthy, deployable tools for health care.

### Conclusions

This study aimed to evaluate trade-offs between classical machine learning and deep learning, including SSL approaches for brain tumor classification under small-scale medical imaging conditions. To achieve this, we compared 4 representative models, including SVM+HOG, ResNet18, ViT-B/16, and SimCLR, with consistent training pipelines and evaluated their performance across both within- and cross-domain settings, without retraining on cross dataset to simulate real-world deployment.

Our analysis revealed that no single model is universally optimal. ResNet18 achieved a strong balance of accuracy and computational efficiency, SimCLR demonstrated superior generalization under limited annotations, ViT-B/16 provided the most coherent visual interpretability through attention mechanisms, and SVM+HOG showed a lightweight, resource-efficient alternative. Importantly, the results highlighted that model choice depended on perspective: from a machine learning researcher standpoint, efficiency and accuracy dominate, whereas from a clinical perspective, interpretability through saliency maps can be important.

## Appendix

Multimedia Appendix 1 Bar charts comparing (top) training and validation accuracy, (middle) training and validation loss, and (bottom) training time across models.

Multimedia Appendix 2 Classification metrics (precision, recall, F1-score, support, macro or weighted, average, and accuracy) for all models on primary validation sets.

Multimedia Appendix 3 Classification metrics (precision, recall, F1-score, support, macro or weighted averages, and accuracy) for all models on primary domain test sets.

Multimedia Appendix 4 Classification metrics (precision, recall, F1-score, support, macro or weighted averages, and accuracy) for all models on cross-domain test sets.

Multimedia Appendix 5 Confusion matrices of the SVM+HOG model evaluated on unseen test data from the (A) primary and (B) cross datasets. HOG: histogram of oriented gradients; SVM: support vector machine.

Multimedia Appendix 6 Confusion matrices of the ViT-B/16 model evaluated on unseen test data from the (A) primary and (B) cross datasets. ViT: vision transformer.

Multimedia Appendix 7 Confusion matrices of the SimCLR model evaluated on unseen test data from the (A) primary and (B) cross datasets. SimCLR: Simple Contrastive Learning of Visual Representations.

Multimedia Appendix 8 Summary of hyperparameter tuning for all models, including tested and final values, and rationale for selected settings across SVM+HOG, ResNet18, ViT-B/16, and SimCLR. HOG: histogram of oriented gradients; SimCLR: Simple Contrastive Learning of Visual Representations; SVM: support vector machine; ViT: vision transformer.

Multimedia Appendix 9 Summary of the overall system configurations across all models.

## Abbreviations

AI: artificial intelligence
CNN: convolutional neural network
GPU: graphics processing unit
HOG: histogram of oriented gradients
MRI: magnetic resonance imaging
SimCLR: Simple Contrastive Learning of Visual Representations
SSL: self-supervised learning
SVM: support vector machine
ViT: vision transformer

## Data Availability

The datasets and model configurations generated and analyzed during this study are available in the Zenodo repository [37]. The source code necessary to reproduce the results is available in the Zenodo repository [38].
