# Supplementary figures and images for: Trade-Off Analysis of Classical Machine Learning and Deep Learning Models for Robust Brain Tumor Detection: Benchmark Study
Source: JMIR AI. 2025 Sep 15;4:e76344. doi: 10.2196/76344 (PMC12456844; doi:10.2196/76344)

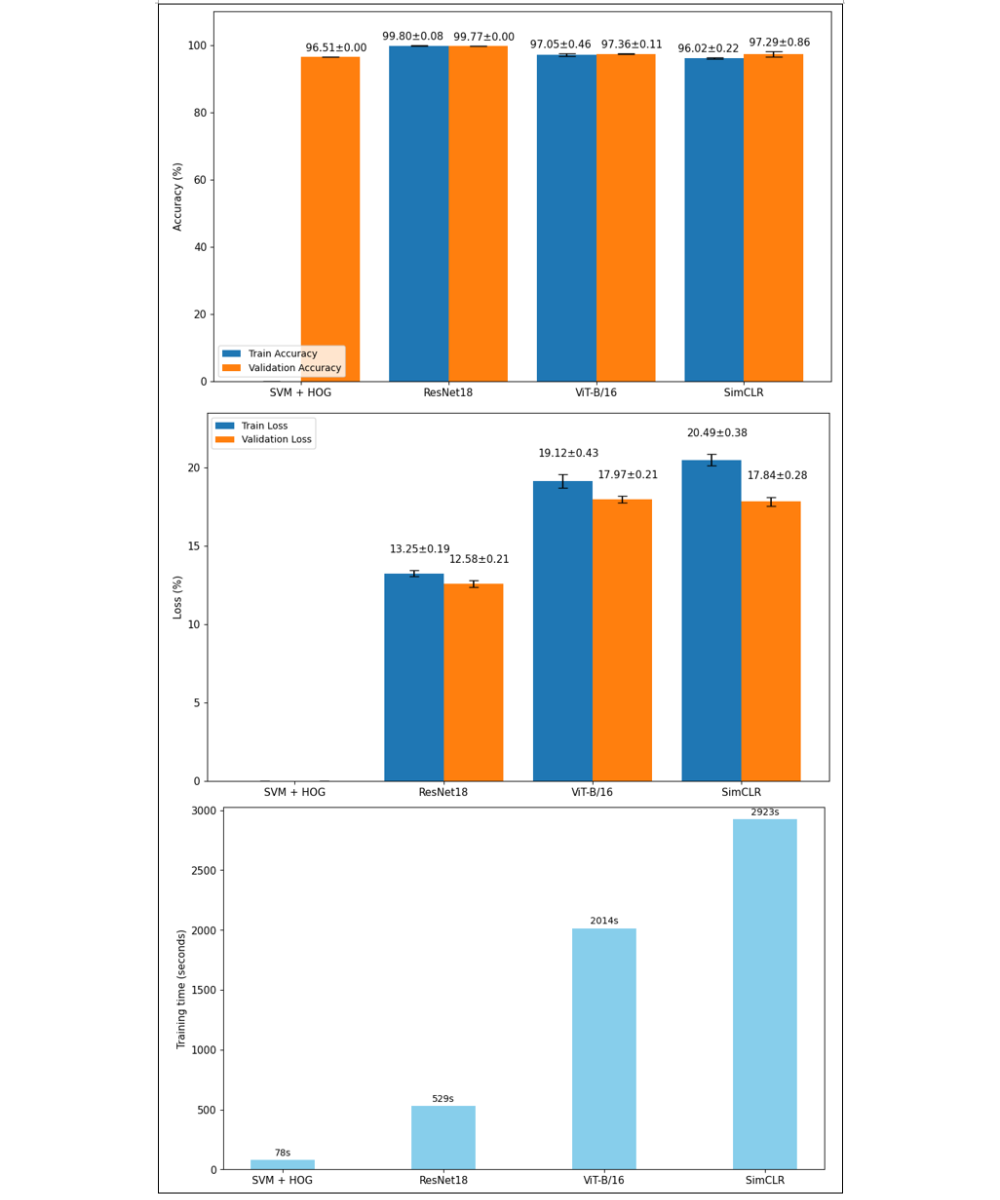

Supplement: Multimedia Appendix 1 [file ai_v4i1e76344_app1.png]

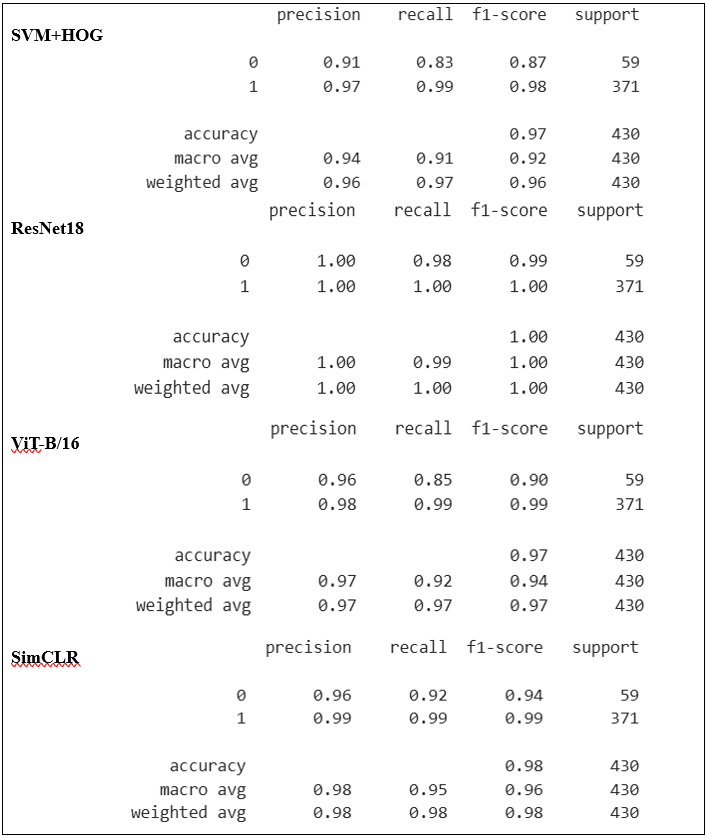

Supplement: Multimedia Appendix 2 [file ai_v4i1e76344_app2.png]

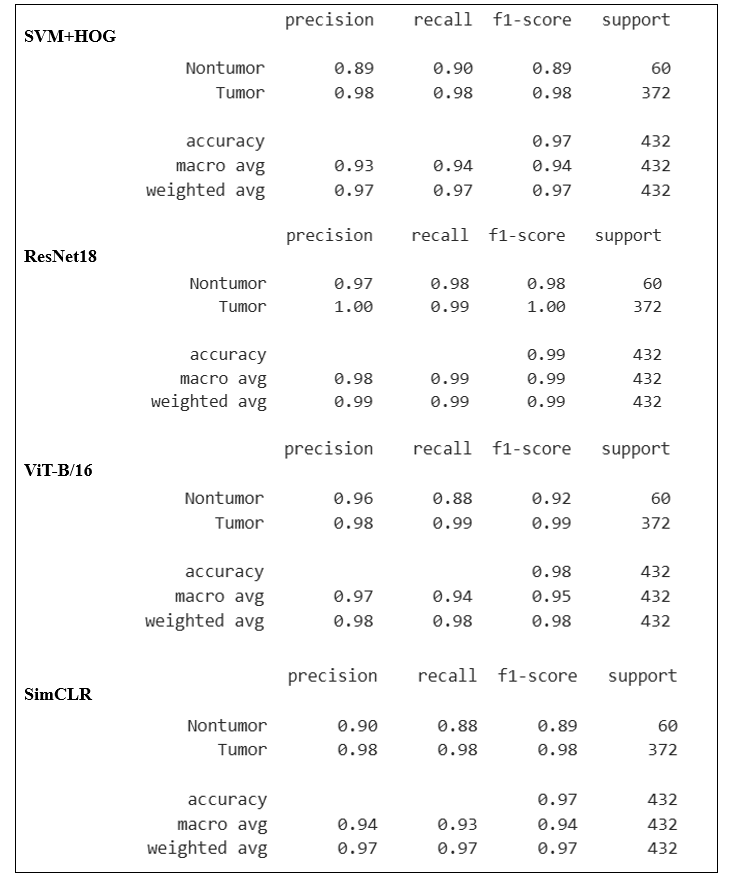

Supplement: Multimedia Appendix 3 [file ai_v4i1e76344_app3.png]

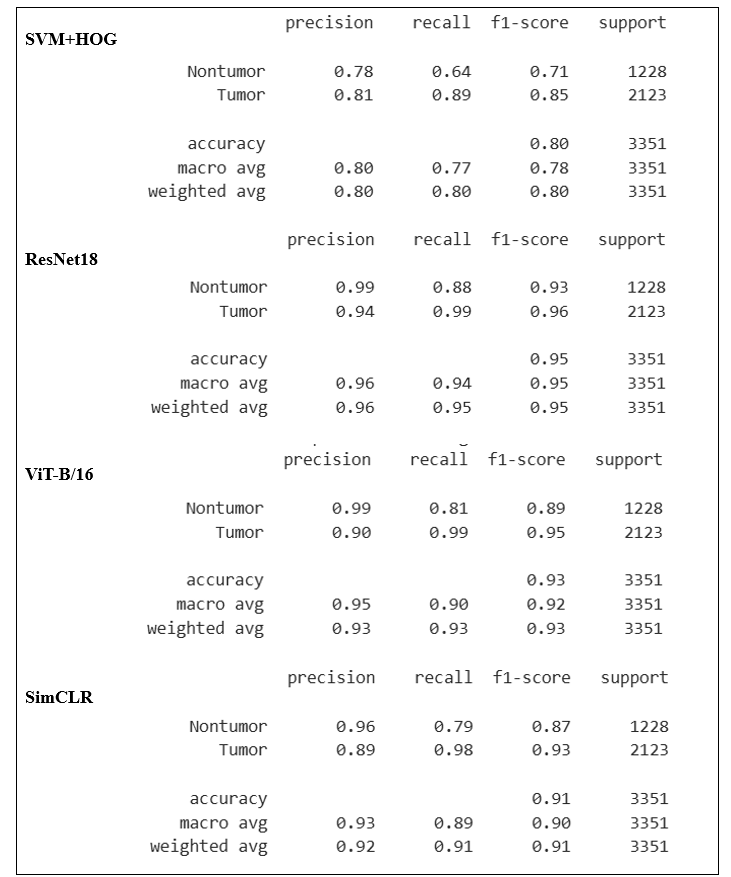

Supplement: Multimedia Appendix 4 [file ai_v4i1e76344_app4.png]

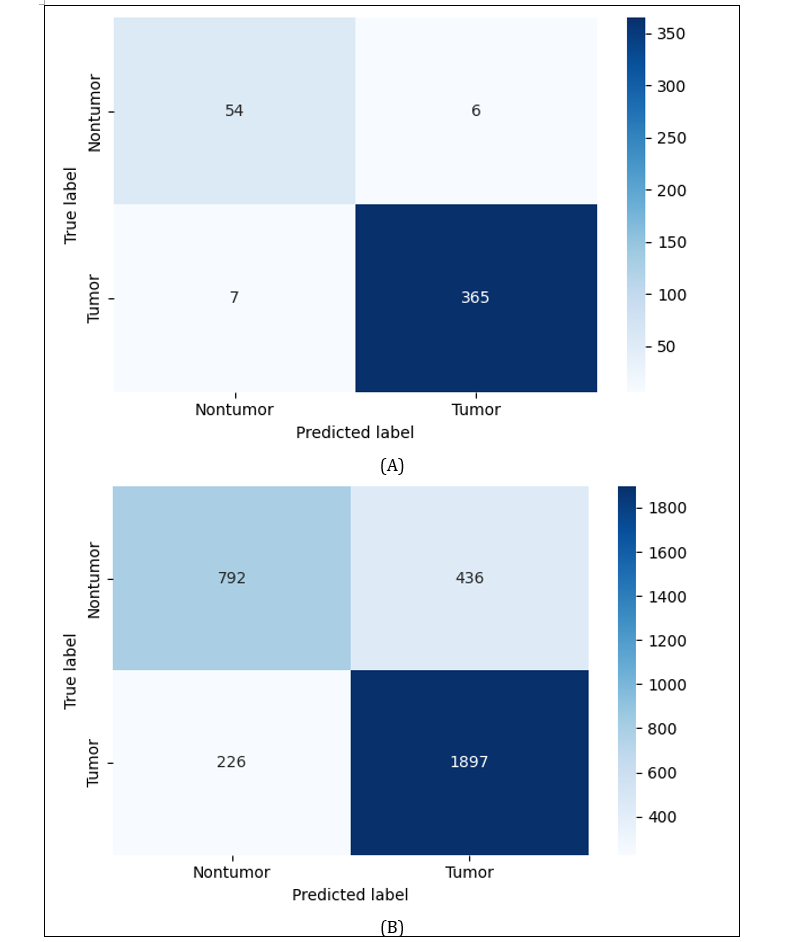

Supplement: Multimedia Appendix 5 [file ai_v4i1e76344_app5.png]

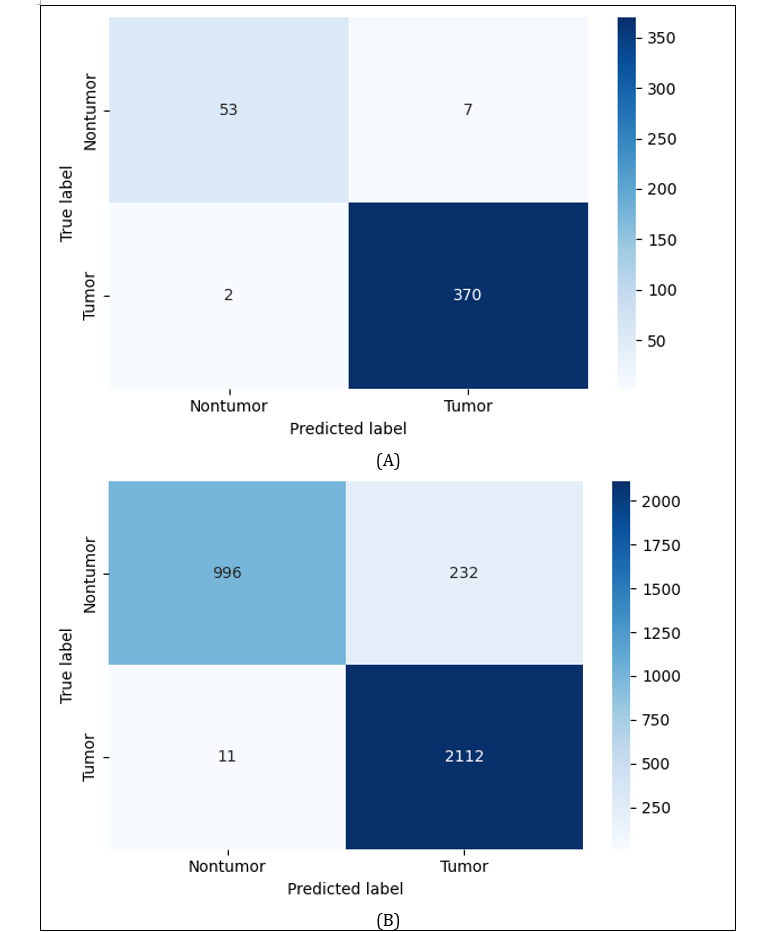

Supplement: Multimedia Appendix 6 [file ai_v4i1e76344_app6.png]

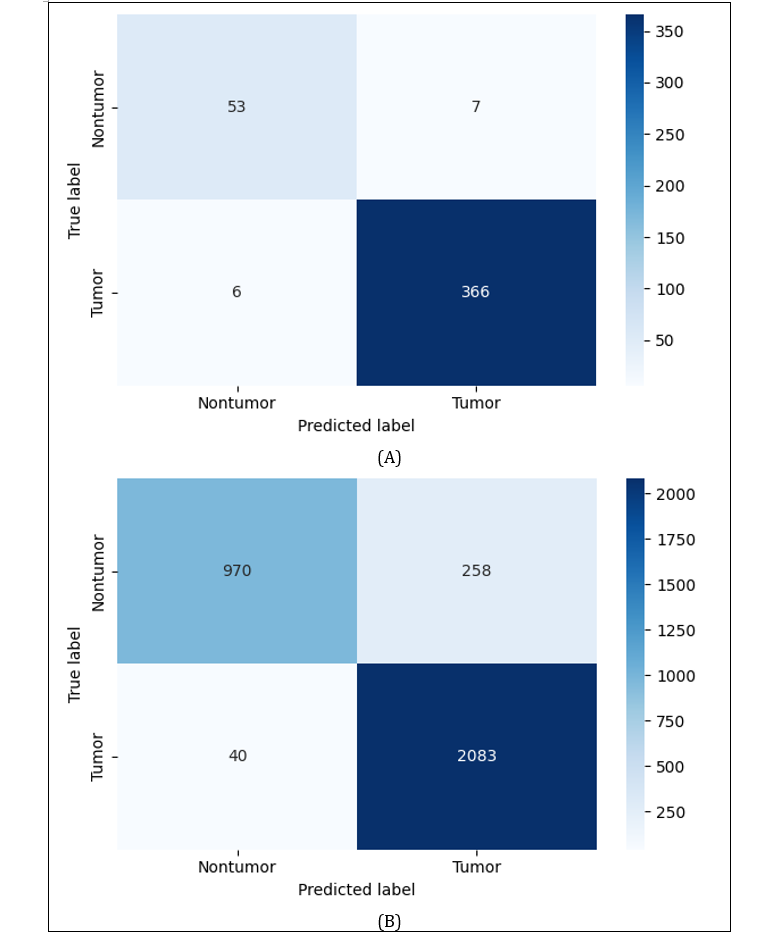

Supplement: Multimedia Appendix 7 [file ai_v4i1e76344_app7.png]

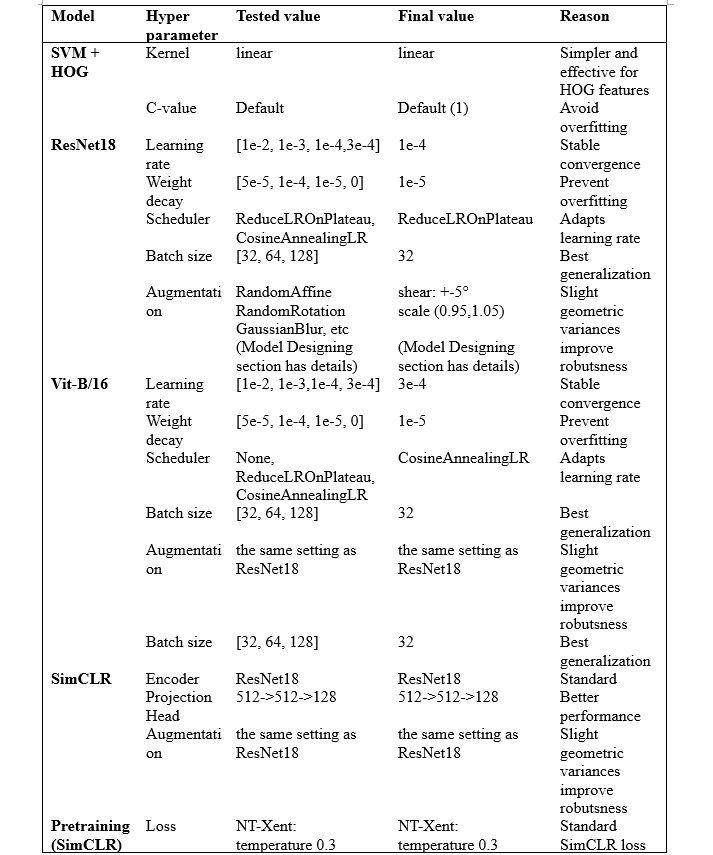

Supplement: Multimedia Appendix 8 [file ai_v4i1e76344_app8.png]

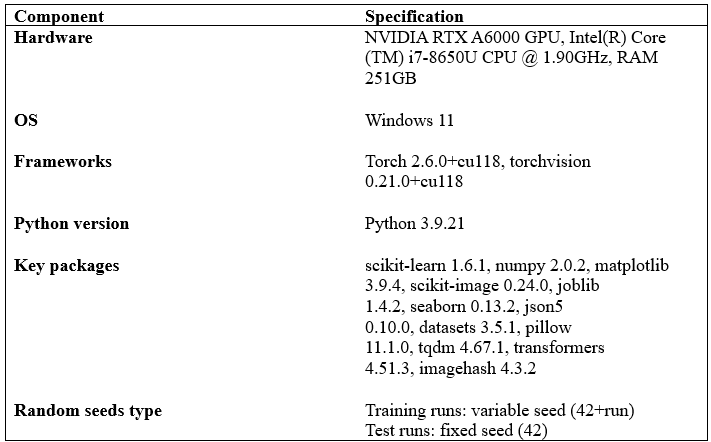

Supplement: Multimedia Appendix 9 [file ai_v4i1e76344_app9.png]
